# Book Review: Pocketbook of Clinical IR

**DOI:** 10.5334/jbsr.1823

**Published:** 2019-05-13

**Authors:** Tushar Garg

**Affiliations:** 1 Seth G.S. Medical College and K.E.M. Hospital, IN

**Keywords:** Pocketbook, IR, Education

## Abstract

Pocketbook of Clinical IR is a skilfully organised pocketbook which is primarily targeted towards medical students. It focuses mainly on clinical care associated with the procedure.

*Pocketbook of Clinical Interventional Radiology (IR)* (Figure 1) is a skillfully organized pocketbook, primarily targeted towards medical students. It focuses mainly on clinical care associated with the procedure.

**Figure 1:**
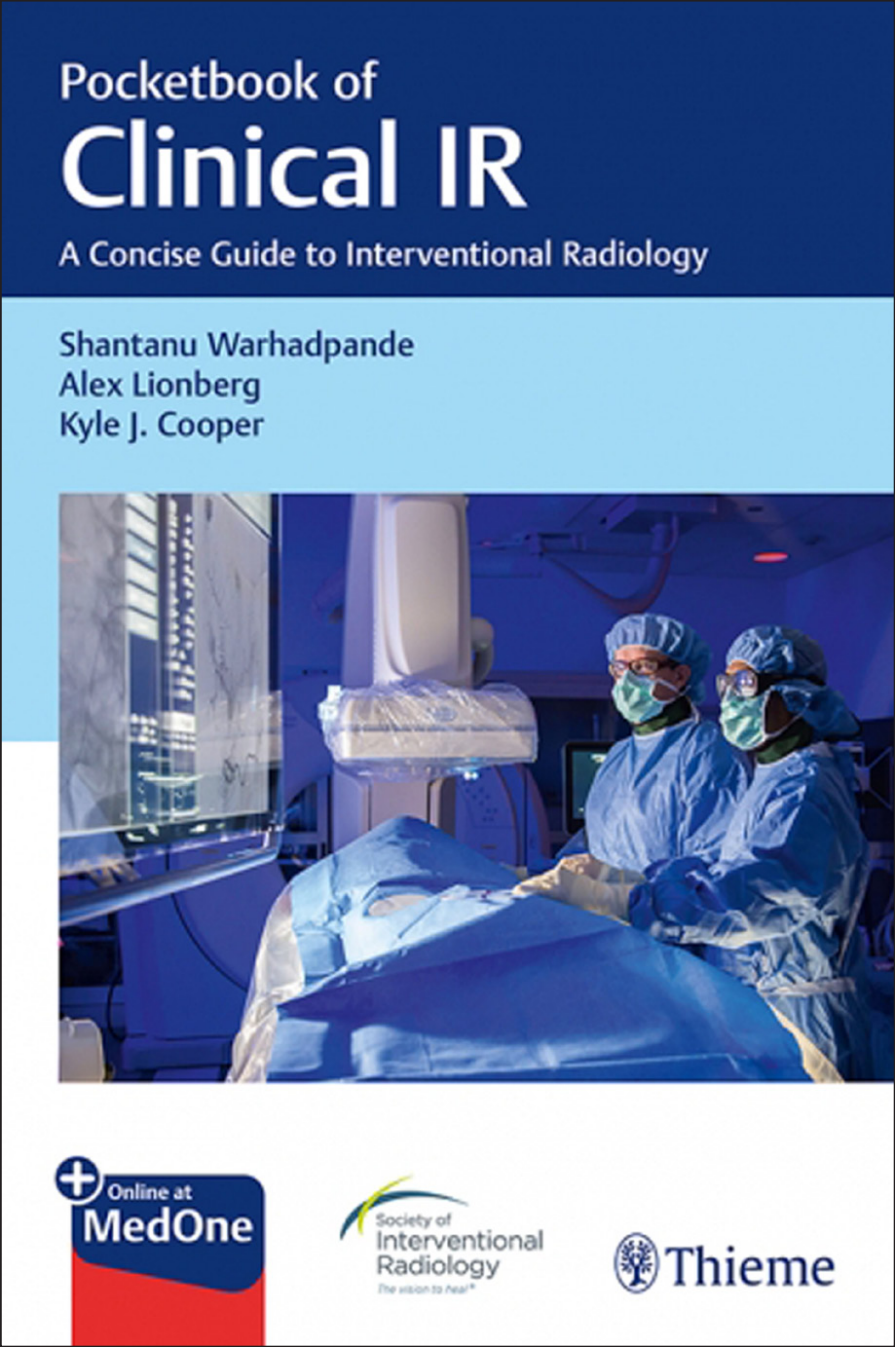
Book Cover.

The first four chapters of the book deal with the basic details that any trainee in IR should be aware of. They address topics such as pre- and post-procedure management, types of vascular and non-vascular procedures, sites and methods for vascular access, and the drains, lines, and tubes that are commonly used in procedures such as venous access and fluid drainage.

After covering the basics, the book delves into the clinical sub-specialties with separate chapters on emergency IR, hepatobiliary interventions, interventional oncology, vascular interventions, genitourinary, neuro IR, and pediatric IR. The special emphasis placed by the author on the process of decision making around a procedure is phenomenal.

The inclusion of “procedure boxes” is another tremendous addition to the book, as it helps the trainee to gain a basic framework about the procedure in a short amount of time. The book in smaller in size compared to its counterparts, but it covers almost all the commonly seen cases along with their basic pathophysiology and management.

I think any student who is interested in or would like to pursue IR in the future should read this book as it provides a solid foundation to build upon.

Reference of the book: https://www.thieme.com/books-main/radiology/product/5185-pocketbook-of-clinical-ir;

Edition: 1
Pages: 240
Illustrations: 165
E-book: €29.99
Print and E-book Bundle: €49.99

